# Supplementary material for: Changes in Content of Polyphenols and Ascorbic Acid in Leaves of White Cabbage after Pest Infestation
Source: Molecules. 2019 Jul 18;24(14):2622. doi: 10.3390/molecules24142622 (PMC6680958; doi:10.3390/molecules24142622)
Supplement: Supplementary file 1 [file molecules-24-02622-s001.pdf]

Changes of polyphenols and ascorbic acid content in leaves of white cabbage after pest infestation

Zuzana Kovalikova [1*], Jan Kubes [2], Milan Skalicky [2*], Nikola Kuchtickova [1], Lucie Maskova [1], Jiri Tuma [1], Pavla Vachova [2] and Vaclav Hejnak [2]

[1] Department of Biology, Faculty of Science, University of Hradec Kralove, Rokitanskeho 62, 500 03, Hradec Kralove, Czech Republic; zuzana.kovalikova @uhk.cz (Z.K.); nikola.kuchtickova@seznam.cz (N.K.); lucina.maskova@seznam.cz (L.M.); jiri.tuma@uhk.cz (J.T.)

[2] Department of Botany and Plant Physiology, Faculty of Agrobiology, Food and Natural Resources, Czech University of Life Sciences Prague, Prague, Czech Republic; kubes@af.czu.cz (J.K.); skalicky@af.czu.cz (M.S.); hejnak@af.czu.cz (V.H.); vachovap@af.czu.cz (P.V.)

* Correspondence: skalicky@af.czu.cz; zuzana.kovalikova@uhk.cz; Tel.: +420-224-382-520

Table S1. The content of observed stress compound, primary and secondary metabolites in control sample of white cabbage extract.

Compound	Superoxide [μg·g^{-1} FW]	Total soluble proteins [mg·g^{-1} FW]	Phenylalanine [nmol·g^{-1} FW]	Tyrosine [nmol·g^{-1} FW]	Total phenoles content [mg·g^{-1} FW]	Total flavonoid content [mg·g^{-1}FW]
	9.778 ± 0.325	18.778 1.859	112.440 8.504	110.717 1.354	3.573 0.115	2.754 0.125

Data are mean ± SE of three repeats.

Table S2. The content of observed phenolic acids in control sample of white cabbage extract.

Compound	*t*-cinnamic	*p*-coumaric	caffeic	ferulic	chlorogenic	benzoic	Salicylic [mg·g^{-1}DW]	4-hydroxy benzoic
	2688.357 ± 1033.640	4724.572 ± 456.100	83056.360 ± 28086.008	16060.891 ± 602.635	561915.499 ± 40482.49	5599.755 ± 482.7370	110.089 ± 12.784	19075.611 ± 2282.984

Data are mean ± SE of three repeats.

Table S3. The content of observed flavonoids in control sample of white cabbage extract.

Compound	quercetin	luteolin	kaempferol
	87.870 ± 5.435	316.561 ± 50.058	441.624 ± 116.531

Data are mean ± SE of three repeats.
